# Ontologies and Knowledge Graphs in Oncology Research

**DOI:** 10.3390/cancers14081906

**Published:** 2022-04-10

**Authors:** Marta Contreiras Silva, Patrícia Eugénio, Daniel Faria, Catia Pesquita

**Affiliations:** LASIGE, Faculdade de Ciências da Universidade de Lisboa, Campo Grande, 1749-016 Lisboa, Portugal; peugenio@lasige.di.fc.ul.pt (P.E.); dpfaria@fc.ul.pt (D.F.); clpesquita@fc.ul.pt (C.P.)

**Keywords:** ontologies, semantic technologies, knowledge graph, cancer, oncology, review

## Abstract

**Simple Summary:**

Cancer is a complex phenomenon and cancer research is increasingly data-rich. Representing this knowledge in a manner that is both human and computer-friendly can help manage and analyze the high volumes of complex cancer data that are created by scientific research and health care. This review looks at the last decade of works on using ontologies—computational representations of knowledge—in cancer, describing their contributions and achievements and charting a path for future research in this area.

**Abstract:**

The complexity of cancer research stems from leaning on several biomedical disciplines for relevant sources of data, many of which are complex in their own right. A holistic view of cancer—which is critical for precision medicine approaches—hinges on integrating a variety of heterogeneous data sources under a cohesive knowledge model, a role which biomedical ontologies can fill. This study reviews the application of ontologies and knowledge graphs in cancer research. In total, our review encompasses 141 published works, which we categorized under 14 hierarchical categories according to their usage of ontologies and knowledge graphs. We also review the most commonly used ontologies and newly developed ones. Our review highlights the growing traction of ontologies in biomedical research in general, and cancer research in particular. Ontologies enable data accessibility, interoperability and integration, support data analysis, facilitate data interpretation and data mining, and more recently, with the emergence of the knowledge graph paradigm, support the application of Artificial Intelligence methods to unlock new knowledge from a holistic view of the available large volumes of heterogeneous data.

## 1. Introduction

Understanding complex phenomena that cannot be modeled purely mathematically is a challenging endeavor transverse to all biomedical research. Ultimately, all boils down to the complex interplay between genes and environment, which manifests in the interactions between the cells in an organism, between host and pathogen, between drug and body. From its genesis, medicine focused on understanding the phenomena which can be generalized between individuals, dating back to the first texts on anatomy by the Ancient Egyptians. Indeed, nomenclature and classification are the first steps towards understanding complex phenomena, and are inextricable from modern medicine, which relies on its precise terminology and its compendium of pathogens, diseases, symptoms, genes and mutations, and drugs and therapies, as well as of the relationships between them.

Over the last three decades, the rise of the digital age and subsequent informatization of clinical records and biomedical research drove the encoding of terminologies, classification schemes and knowledge models into digital machine-readable formats (often captured under the umbrella term ‘ontology’) to promote standardization, support information systems, and enable knowledge discovery. One of the first major efforts to this effect in the biomedical domain was the compilation of the Systematized Nomenclature of Medicine—Clinical Terms (SNOMED CT) [1] to support the standardization and interoperability of clinical information systems and electronic health records. Another major effort was the classification and trans-species standardization of gene functional characteristics under the Gene Ontology (GO) [2]. In the footsteps of these efforts, several hundred other ontologies have been developed for the biomedical domain throughout the years [3], among which we must note the National Cancer Institute Thesaurus (NCIt), a compendium of terminology spanning all aspects of cancer research and health care [4].

More recently, medicine has been witnessing a shift towards the particular, enabled by the decreasing costs of acquiring genetic information, and driven by the understanding that tailored treatments that contemplate the genetic makeup of the patient will likely be more effective and less prone to nefarious side-effects. Cancer is the family of diseases that is benefiting from these precision (or personalized) medicine approaches the most, as despite commonalities, each cancer is genetically unique, and can react very differently to different types of treatment. Moreover, understanding the fine differences between cancer cells and healthy cells can be the key for more successful and less aggressive treatments. Yet the precision medicine paradigm places additional emphasis on having a holistic understanding of the gene–environment interplay in all its manifestations, which requires the integrative analysis of large volumes of heterogeneous data that are individually already complex (e.g., clinical records, medical imaging, transcriptomic data, immunopeptidomic data) [5]. Here too, ontologies have been playing an important role in enabling data integration and facilitating data analysis.

In this article, we review the applications of ontologies in cancer research over the past decade, summarizing published works within this time frame, and categorizing them with respect to their usage of ontologies. Section 2 details core concepts underlying this review article, Section 3 outlines the methodology adopted to conduct the review, Section 4 summarizes both the ontologies reused in the works and the ones created for them, Section 5 reviews and categorizes the aforementioned published works, and Section 6 features our prospects regarding the present and future use of ontologies in cancer research.

## 2. Background

### 2.1. Ontologies

The term “ontology” was borrowed from philosophy to computer science to signify a machine-readable formalization of a conceptualization pertaining to a particular domain of knowledge [6]. That is to say, an ontology is a digital artifact that can be interpreted by both humans and computers and which encodes the terminology and the semantic relations between concepts in a given domain. The term “ontology” is often used with some latitude, also encompassing thesauri [7]. While our review of published works adopts the same encompassing perspective, it is important to make a formal distinction between ontologies proper and thesauri due to their different purposes and applications.

Ontologies proper are typically encoded in the Web Ontology Language (OWL), developed by the W3C OWL Working Group [8], which includes various serializations, namely the Open Biomedical Ontologies (OBO) format or the more popular Resource Description Framework (RDF) format in which statements take the form of triples of the form <subject> <predicate> <object>. OWL defines several types of entities which can be used in constructing ontologies, such as: classes, datatypes, object properties, data properties, annotation properties, individuals and literals, among others. All entities in an ontology are identified by an International Resource Identifier (IRI), although in OBO ontologies this is abbreviated to an alphanumeric code. Annotation properties (e.g., *label*) are used to describe the entities in the ontology for human readers, and thus, encode the terminological component of ontologies; they have no semantic value. Individuals (or instances) and literals are data-level entities representing, respectively, concrete objects (e.g., *my heart*) and data values (e.g., “60 beats/min”). The remaining entities are model-level, with classes representing abstract sets of individuals (e.g., *heart*), datatypes representing abstract sets of literals (e.g., *string*), object properties representing relations that can be used to connect individuals (e.g., *part of*) and data properties representing attributes that can be used to describe individuals with literals (e.g., *has heart beat*). Moreover, OWL defines intrinsic properties that can be used to connect classes (*subclass*, *disjoint*), to assert that individuals belong to a class (*type*), or to constrain object or data properties with respect to the classes that can have them as subjects (*domain*), the classes or datatypes they can take as objects (*range*), or their usage and logic (e.g., *transitive*, *symmetric*). Finally, OWL enables the definition of class expressions, which are classes defined semantically, for example through application of logical operators (*union*, *intersection*, *not*) between classes, or through existential, universal or cardinality restrictions on objects or data properties (e.g., *part of* some *chest*, which can be applied to class *heart*). OWL ontologies have different degrees of expressiveness depending on which of these features they use, ranging from simple class hierarchies up to semantically intricate knowledge models, which has implications on the possible applications of ontologies. Namely, OWL supports deductive reasoning, that is to say, the use of logical inference to derive non-stated facts from the collection of facts explicitly asserted in the ontology, which will be both harder and more likely to result in non-evident facts the more expressive the ontology is.

**Figure 1:**
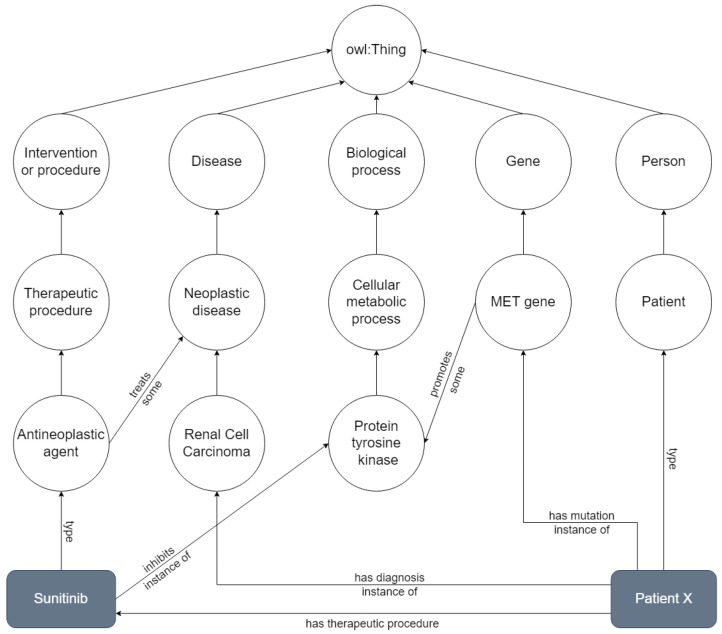
Knowledge graph representing a smaller network that includes renal cell carcinoma, MET gene, antineoplastic agent and proten tyrosine kinase, with instances of a Patient X and the drug Sunitinib. All concepts are derived from the class owl:Thing. Adapted from the NCIt.

Ontologies are often published with only the model-level layer, serving as knowledge models for a given domain, without any data. In some cases, ontologies are used to annotate external data, such as text documents or database entries, without actually instantiating the ontology (e.g., the Gene Ontology is used to annotate genes and proteins, but these are not individuals of the ontology). In other cases, ontologies are developed (or adopted) to serve as the semantic backbone for describing data in a machine interpretable form. When a large number of individuals is represented in a graph that employs an ontology as its schema, we can consider it a Knowledge Graph (KG) [9]. Figure 1 depicts a simplified example of a KG, based on NCIt. Classes are represented as circles in a descending hierarchy stemming from the superclass “owl:Thing”, class instances as grey rectangles, and relationships between them are depicted as arrows, corresponding to object properties in an ontology. This KG shows the network around the concepts renal cell carcinoma, MET gene, antineoplastic agent and protein tyrosine kinase, with instances of patient (“Patient X”) and antineoplastic agent (“Sunitinib”).

Thesauri are much simpler than ontologies, and are typically encoded in the Simple Knowledge Organization System (SKOS), which, curiously, is defined on top of OWL. In SKOS, there is no data-level layer, only a model-level layer comprised of *concepts*, their terminological characterization through annotations, and the loose semantic relations between them (*broader*, *narrower*, *related*). Thus, thesauri are almost exclusively terminological, and do not enable many of the more sophisticated applications of ontologies proper, namely applications that involve reasoning.

### 2.2. Ontologies in Cancer Research

The ability to model complex domains is the reason why ontologies are suitable for cancer research and healthcare. For an especially complex disease, such as cancer, that tends towards individual uniqueness and is comprised of various factors and variables, the ability to represent it fully in a manner that can be understood by both clinicians and researchers, and machine algorithms, is invaluable. As such, ontologies represent a unique opportunity to support the domain complexity while allowing for the construction of equally complex solutions that further aid in diagnosing and treating cancer.

At present, there are numerous publicly available biomedical ontologies that have as their principal aim the description of cancer and its characteristics. The National Cancer Institute Thesaurus (NCIt) is perhaps the most often seen. Additionally, there are other biomedical ontologies that, while not directly related to the subject of cancer, are invaluable in its research, for describing fundamental concepts of biology and medicine that form a solid base on which further information stands. Of these, the Gene Ontology (GO) is the most commonly used.

Ontologies in cancer research can be used in varied manners with differing focal objectives. First, despite the fact that cancer-focused ontologies already exist, further conceptualizations of the domain can be developed in the form of new ontologies [10]. These can be reformulations of actual ontologies, updated to include more entities, or even a new, original, ontology to establish a previously less explored section of knowledge. Furthermore, ontologies can be used to annotate data and connect it to the overall context of the domain it pertains to [11]. In this way, for instance, a single value is not simply an isolated value, it is now a single result value from an RNA Sequencing experiment that is placed in a particular section of biomedical knowledge and holds specific relationships to the remaining domain. This annotated value can then be further integrated into developing solutions and their overall context. In addition, ontologies can be directly used as vocabularies to support the organization of data according to known domain information [12]. One objective for this use is, for instance, allowing users to search data that has been annotated using ontologies in a database. Furthermore, NLP methods also need a comprehensive set of terms to use in their application, that then allows for the identification of this information in long-form text, for example [13]. Due to their axiom-based structure, ontologies can support reasoning applications, first to confirm consistency in the ontology and data themselves [14] but also to obtain further inferences from the formal definitions that are established by the ontologies [15]. Lastly, annotation of data with ontologies allows for further use in mining and analyzing this data, for example, with enrichment methods or similarity measures [16,17]. Additionally, there has been an increase in the use of ontology-structured data as input for ML methods, particularly in the biomedical domain with, for example, gene function predictions and clinical decision support systems [18,19].

## 3. Materials and Methods

### 3.1. Initial Search and Screening

We carried out an initial search of PubMed [20] on 10 January 2022 with the search query: *(“ontology” OR “knowledge graph”) AND “cancer”*. We restricted the search to open access articles between between 2012 and 2021, setting the search to both *Title* and *Other Term*, and in the case of the “cancer” query, additionally also *MeSH Terms*. We complemented this initial search with a search of Google Scholar [21] on 21 March 2022 with the search query: *(“ontology” OR “knowledge graph”) AND (“cancer” OR “oncology”)*. The search was constrained to only the title and between 2012 and 2021. The combined results of the two searches were 360 articles.

**Figure 2:**
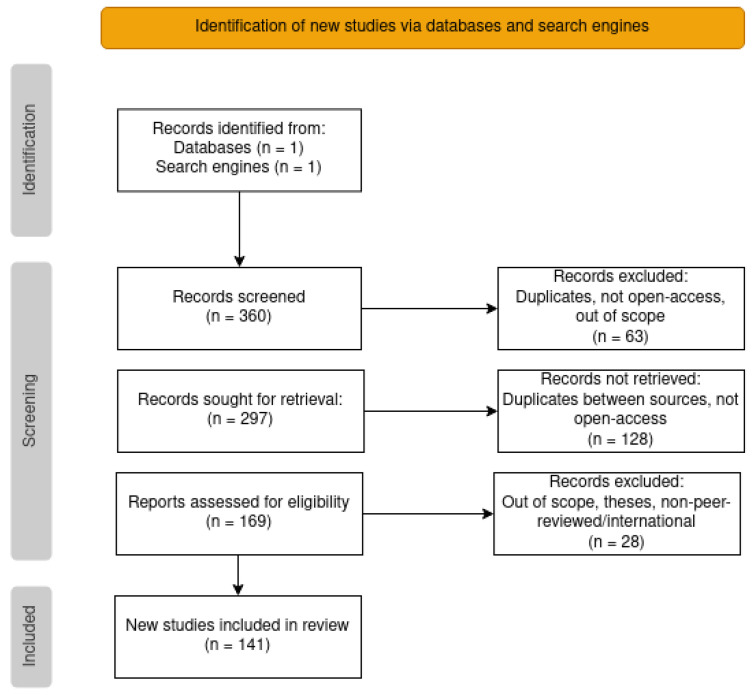
PRISMA flowchart with the steps taken to reach the final list of articles for categorization.

We screened the resulting lists of articles with the following exclusion criteria: duplicated articles, non-open access articles, and out of scope articles. The latter encompassed articles not related to cancer (misclassification, typos such as oncology/ontology, or mention of only cancer cell lines but not to cancer), articles which did not clearly describe the use of ontologies, and review articles. Additionally, from the Google Scholar results we also excluded theses and non-international and/or non-peer-reviewed publications (which were not an issue in the PubMed search). The screening was conducted in stages, by first examining the title and accessibility of the article, then reading the abstract, and finally reading the article in its entirety. From the initial list of 360 articles, the screening resulted in only 141. A workflow diagram of the whole process can be viewed in Figure 2.

### 3.2. Categorization

We developed a novel categorization scheme composed of 14 hierarchical categories that describe how the reviewed works employ ontologies and knowledge graphs. These categories fall into two main branches: *Terminology-focused applications* and *Semantic-focused applications*.

The original purpose of clinical and biomedical ontologies was to serve as a source of controlled terminology to tackle the challenges of data-intensive research and clinical practice. As biomedical data production increases and the further it spreads across databases and repositories, there is a reinforced need to connect it to the overall context and to assign the same “meaning” to data that is saved in different and independent places. Ontologies represent the domain concepts in a standardized manner—using a unique identifier for each concept—and placing data into this context increases its own individual reusability by ensuring that it will be understood by anyone, but also, it allows for data from different sources to be easily matched in their relation to a specific entity.

We have organized **Terminology-focused applications** under four categories:

- **Data Annotation:** ontologies are used to describe data under a common schema, linking data objects to ontology classes that describe them.
- **Data Integration:** ontologies support the integration of different data sets or databases.
- **Database Interface:** ontologies are used to support user interfaces for databases, where labels of ontology classes and relations allow text annotation. These interfaces are notably useful in dealing with medical data, for integration and querying of different knowledge resources.
- **NLP:** ontologies are used as the vocabulary source for Natural Language Processing (NLP) methods, where entities, events or relations in a text are identified through the corresponding ontology labels.

**Semantic-focused applications** fall under two sub-categories, which are further subdividided:

- **Reasoning:** Automatic reasoners process ontologies’ axioms and their formal definitions.
  – **Inference of New Knowledge:** complex reasoning-based queries can reveal novel biological knowledge based on the already defined axioms.
  – **Error Detection:** reasoning applied to check for consistency (or contradictions) in the ontology.
- **Data Mining and Analytics:** ontologies are used to support data mining and analytics tasks.
  – **Semantic Filtering:** ontology-based annotations are used to filter and process data.
  – **Semantic Similarity:** ontology-based annotations are used to compare data entities.
  – **Machine Learning:** ontologies and KGs are explored by machine learning algorithms.
  – **Gene Set Enrichment:** statistical analysis of gene set ontology-annotations.

From the final list, articles were sorted into one or more of the 10 leaf categories according to how the work uses ontologies.

**Figure 3:**
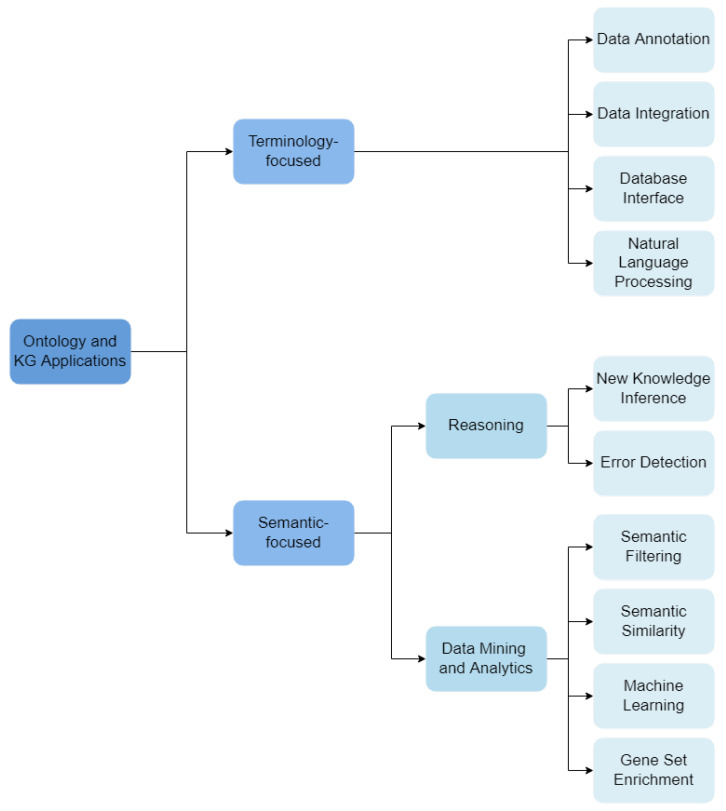
Classification schema for the works included in this articles.

The schema of classification is shown in Figure 3, outlining all the categories and their hierarchical organization used in the following sections.

## 4. Ontologies in Oncology

### 4.1. Ontologies Used in the Reviewed Applications

One of the ontologies most commonly used in cancer research is, as expected, the National Cancer Institute thesaurus (NCIt) [22], which is a comprehensive ontology devoted specifically to cancer and encompassing both the clinical and research aspects. The SNOMED-CT [1], a broad scope healthcare ontology that has played a key role in systematizing electronic health records, has been used in applications involving clinical data. UMLS [23] is also popular, and is the largest compendium of biomedical terminology, aggregating several healthcare ontologies and vocabularies (namely NCIt and SNOMED-CT) and including mappings between them to enable interoperability.

The Medical Subject Headings (MeSH) thesaurus [24], which are used to index scientific publications, have often been used for bibliographic searches and natural language processing applications. The Disease Ontology (DO) [25] is narrower in scope than the UMLS, focusing only on diseases, but also includes extensive mappings to other healthcare vocabularies (namely MeSH, NCIt and SNOMED-CT).

Other ontologies with narrower scope nevertheless describe aspects that are critical for cancer research. Among them, we include the oncology subset (ICD-O) of the International Classification of Disease (ICD) [26], which categorizes tumors; the Ontology for Biomedical Investigations (OBI) [27], which aims to describe the terms related to biological and medical investigations; the Cell Line Ontology [28] which classifies cell lines; the Time Event Ontology (TEO) [29], which models temporal expressions and is especially useful when dealing with timed occurrences as healthcare often includes; and the Gene Ontology (GO) [2], which describes gene functions. The latter is the most used ontology of the works reviewed, as it is employed in almost all Gene Set Enrichment applications.

### 4.2. Ontologies Created for the Reviewed Applications

Several works pertaining to ontologies in cancer research reported on the creation of new ontologies, as summarized in Table 1. The fact that multiple ontologies have been developed in this domain reflects the fact that an ontology is a conceptualization formalized for a particular objective, which represent a given point of view of the underlying domain. As such, despite the existence of several ontologies within the domain, it is often necessary to develop new ontologies for different purposes or to model novel datasets. This is also a testament to the complexity of the cancer research domain, and the several biomedical disciplines it traverses.

One common reason why new ontologies have been developed was to semantically formalize already existing standards. Within this category, Nicholson et al. [30] derived the ENCR core-data ontology from the European Network of Cancer Registries (ENCR) data-validation rules to further support the validation of cancer datasets through an unambiguous formalization and ensure coherence through automatic reasoning logic. Similarly, Zhang et al. [31] also developed the Ontology for the Documentation of vAriable selecTion and daTa sourcE Selection and inTegration (OD-ATTEST) based on a set of reporting guidelines for cancer risk factor variable and data source selection to serve as a standardization of data models. With the aim of describing cancer cells and capturing the properties of tumorigenesis, Rasmussen et al. [32] created the OncoCL. Jusoh et al. [33] built a breast cancer ontology using a hybrid approach to help integrate cancer data from different sources into a single database. Furthermore, in the breast cancer domain, Myneni et al. [34] created OntoMama to assist medical students and professionals. Malty et al. [35] created an ontology of standardized cancer treatments that maps to standard nomenclatures based on HemOnc. Dinakarpandian et al. [36] created the Temporal Ontology for Comparing the Survival Outcomes (TOCSOC), a temporal ontology of survival outcome measures of clinical trials in oncology, reusing numerous ontologies. PCLiON is a new standardized lifestyle ontology created by Chen et al. [37], reusing multiple ontologies to harmonize the different data types related to prostate cancer. Looking to generalize the pattern of definitions to correctly classify all gastrointestinal tumor configurations, Herrmann et al. [38] developed their ontology based on BioTopLite2.

Another common reason for ontology development is to create a semantic model for existing datasets. For example, Esteban-Gil et al. [39] used data from a cancer registry relational database to develop a semantic model that can then be queried to analyze patient data through ontology-driven search. The NeoMark European project [11] also developed a specialized ontology for their data content, the NeoMark Ontology, built from its existing database. Amith et al. [40] used a lightweight Open Information Extraction (OIE) tool to extract semantic information from MedlinePlus and seed a knowledge-base. To represent obesity related cancer information, organize and allow data querying, Elhefny et al. [41] reused DOID to develop the Fuzzy Ontology for Obesity-Related Cancer (FOORC).

Ontologies have also been developed to harmonize the communication between clinicians and patients, namely by exploiting social media. Tapi Nzali et al. [42] built a Consumer Health Vocabulary (CHV) in french for breast cancer by mapping terms from forum messages and standardized medical terms. Lee et al. [43] created an ontology to understand information needs and emotions regarding cancer from social media. Myneni et al. [34] developed the Profile Ontology for Cancer Survivors (POCS) to facilitate the fast development of patient-engaging mobile apps.

Supporting the development of applications to aid diagnosis and treatment by providing a semantic representation of existing knowledge has been another major motivation for the development of new ontologies. For hepatocellular carcinoma (HCC), Messaoudi et al. [44] developed the Ontology of Hepatocellular Carcinoma (OntHCC) to support their application in the detection of nodules in medical imaging, while Gurcan et al. [45] created the Quantitative Histopathological Imaging Ontology (QHIO) to represent both data and methods used in clinical imaging and analysis. Boeker et al. [46] developed TNM-O to represent the Tumor–Node–Metastasis (TNM) classification of malignant tumors and Tagliaferri et al. [47] developed the ENT COBRA (COnsortium for BRachytherapy data Analysis) ontology to standardize data collection for head and neck cancer patients that have been specifically treated with interventional radiotherapy, while SKIN-COBRA has a similar objective for non-melanoma skin cancer patients with the same treatment [48]. With a very focused aim, Oyelade et al. [14] proposed Breast Cancer Fuzzy Ontology (BCFO) to address vagueness in the domain of this specific cancer. Mahmoodi et al. [49] manually created the Gastric Cancer Ontology (GCO) with experts to support the extraction of association rules. Gao et al. [50] constructed a treatment-based cancer ontology using a Bayesian derivation that focuses on cancer reclassification and drug inference. For lung cancer, Sesen et al. [51] constructed the LUCADA ontology to use with the clinical decision support application Lung Cancer Assistant. In the domain of bladder cancer, Barki et al. [52] developed an ontology to predict side effects caused by treatments.

Finally, ontologies have been developed for enabling data interoperability and integration, a pressing demand given the increasing volume of heterogeneous data sources available for cancer research. To study the connection between various risk factors and cancer survival, Zhang et al. [53] created the Ontology for Cancer Research Variables (OCRV) reusing some existing resources, and then linked it to a data integration pipeline. Lin et al. [10] developed the Cancer Care Treatment Outcome Ontology (CCTOO) that organizes high-level oncology treatment end points into four domains: cancer treatment, health services, physical, and psycho-social health-related concepts. To aid drug target prediction, Tao et al. [54] created the CRC ontology, reusing PharmGKB. Balasubramanian et al. [55] reused BFO and created the Ontology of Cancer Related Social-Ecological Variables (OCRSEV) to enable data integration and posterior association between Social-Economical Factors and health outcomes in cancer. Aiming to increase interoperability between data sources to allow the creation of Big Data studies that involve several treatment centers, Bibault et al. [56] created the Radiation Oncology Structures (ROS) based on FMA. To also support integrative data analysis in cancer outcomes research, Zhang et al. [57] created the Ontology for Documentation of Variable and Data Source (ODVDS) reusing BFO. Divakar et al. [58] developed CCOWL in order to analyze patient’s cytological tissue images of cervical cancer. Additionally, RiskExplorer was created by Daowd et al. [59] to represent causal associations between the incidence of breast cancer and risk factors.

Some works also report on updates or extensions to existing ontologies, motivated by some of the same objectives for creating new ontologies. Serra et al. [60] developed the Cancer Cell Ontology (CCL) as an extension of the Cell Ontology (CL), to serve as a formal representation of immunophenotyping cell types from hematologic malignancies. The Cell Line Ontology (CLO) was updated and extended by Ong et al. [61] to include NIH Common Fund Library of Integrated Network-based Cellular Signatures (LINCS) cell lines, with a subset LINCS-CLOview being generated. Campbell et al. [62] created additional concepts for Systematized Nomenclature of Medicine Clinical Terms (SNOMED CT) that unify it with Logical Observation Identifier Names and Codes (LOINC) for colorectal and breast cancer.

**Table 1:** New ontologies.

Ref	Objective	Ontology Name	Domain	Reused Ontologies	Language
[51]	Model lung cancer for the clinical decision support application Lung Cancer Assistant	LUCADA ontology	Clinical	SNOMED-CT	OWL
[33]	Use a hybrid approach to build a breast cancer ontology	N/A	Breast Cancer	N/A	OWL
[32]	Describe cancer cells and capture the properties of tumorigenesis	OncoCL	Cell Lines	CL, UBERON, BTO, Pathway Ontology, PATO, CPO, SO	OWL
[11]	Represent the project domain and link the NeoMark data to other domains	NeoMark ontology	Clinical	BFO, RO	OWL
[50]	Cancer reclassification and drug inference	N/A	Farmacology	N/A	N/A
[54]	Drug target prediction	CRC ontology	Colorectal Cancer	PharmGKB	OWL
[63]	Assist medical students and professionals in the breast cancer domain	OntoMama	Clinical	N/A	N/A
[34]	Development of an ontology-driven survivor engagement framework for mobile apps	POCS	Social	FOAF	OWL
[46]	Creation of TNM-O	TNM-O	Anatomical	FMA, BioTopLite 2	OWL
[41]	Represent obesity-related cancer (ORC) ontology to organize information and allow data querying	FOORC	Obesity Related Cancer	DOID	OWL
[49]	Extraction of association rules from large datasets on gastric cancer patients	Gastric cancer ontology	Clinical	N/A	N/A
[55]	Aid data integration; enable association between SE variables and health outcomes	OCRSEV	Social-Ecological Factors	BFO	OWL
[45]	Interoperability across quantitative histopathological imaging data sets	QHIO	Imaging	OBI	OWL
[39]	Design of a semantic model for local cancer registries	N/A	Epidemiology	SIO, OBI	OWL
[40]	Development of ontologies for the public health domain	N/A	Public Health	N/A	OWL
[61]	Understand cellular responses to different perturbations	LINCS-CLOview	Cell Lines	CLO	OWL
[53]	Integrate heterogeneous datasets	OCRV	Cancer Outcomes	BFO, NCIt, TEO	OWL
[47]	Define a specific terminological system to standardized data collection for head and neck cancer patients	ENT COBRA ontology	Clinical	N/A	N/A
[10]	Use structured knowledge representation with concepts of treatment end points	CCTOO	Clinical	NCIt, CTCAE	OBO
[62]	Represent the data elements identified by the synoptic worksheets of College of American Pathologists	SNOMED CT observable ontology	Clinical	SNOMED CT, LOINC	N/A
[35]	Create a standardized hierarchic ontology of cancer treatments, mapped to standard nomenclatures	N/A	Cancer Treatments	HemOnc	OWL
[56]	Increase interoperability between data sources to allow the creation of Big Data studies involving several treatment centers	ROS	Radiation Oncology	FMA	OWL
[36]	Create temporal ontology of survival outcome measures of clinical trials in oncology	TOCSOC	Clinical	EFO, CCTOO, IOBC, NCIT	OWL
[60]	Provide an ontological representation of immunophenotyping cell types found in hematologic malignancies	CCL	Hematologic Malignancies	CL	OWL
[42]	Semi-automatic development of CHV for breast cancer	MuEVo	Clinical	MeSH, MedDRA, SNOMEDint	SKOS
[44]	Offer ontology-based approach modeling HCC tumors	OntHCC	Liver Cancer	N/A	OWL
[57]	Support integrative data analysis in cancer outcomes research	ODVDS	Risk Factors	BFO	OWL
[58]	Cytological tissue image analysis of cervical cancer	CCOWL	Cervical Cancer	N/A	OWL
[31]	Standardize the terminology used in the selection and integration steps of RF variables and data sources	OD-ATTEST	Risk Factors	BFO, others in NCBO (not specified)	OWL
[48]	Standardize data collection for non-melanoma skin cancer patients treated with brachytherapy	SKIN-COBRA ontology	Clinical	N/A	N/A
[43]	Analyze social media data to identify information needs and emotions related to cancer	N/A	Social	LCO, BCO, GCO, SOSW	N/A
[37]	Solve the heterogeneity and diversity of different data types related to prostate cancer by establishing a standardized lifestyle ontology	PCLiON	Risk Factors	NCIT, WordNet, SNOMED CT, The Cochrane Library, FooDB, CheBI	OWL
[59]	Build a knowledge graph that represents causal associations between incidence of breast cancer and risk factors	RiskExplorer	Clinical	UMLS	N/A
[30]	Facilitate the integrity and maintenance of ENCR core data set.	ENCR core-data	Epidemiology	N/A	OWL
[14]	Minimizing vagueness in the formalization of medical knowledge	BCFO	Clinical	DO	OWL
[52]	Predict side effects of bladder cancer treatments	N/A	Bladder Cancer	N/A	OWL
[38]	Provide a generalizing pattern of more concise definitions to correctly classify all tumor configurations	N/A	Gastrointestinal Tumors	BioTopLite2	N/A

## 5. Ontologies and Knowledge Graph Applications in Cancer Research

The categorization of the reviewed works relied exclusively on the information presented in the article and no additional searches were conducted to obtain further details. The information gathered in the process of categorization is presented in Table 2, Table 3 and Table 4 organized into columns relevant to each category.

### 5.1. Terminology-Focused Applications

Table 2 describes the articles from these categories, according to the ontologies and data employed and cancer type.

#### 5.1.1. Data Annotation

Most *Data Annotation* works use existing ontologies, such as NCIt, Medical Subject Headings (MeSH), and GO, among others, but there are quite a few instances where new ontologies were created to address specific needs.

In breast cancer, Zhu et al. [15] used the semantic modeling of drugs from PharmGKB to infer repositioning. As cancer care is a continuum, Myneni et al. [34] developed an ontology-driven adolescent and young adult survivor engagement framework, to aid the development of mobile apps for information dissemination about treatments and effects of cancer therapies provided through Survivorship Care Plans. Esteban-Gil et al. [39] created a semantic representation of data from a cancer registry database, that results in a model that can be reused and extended to other registries and is capable of supporting further semantic queries on patient profiles that are crucial to research. Yan et al. [13] used NLP tools and an enriched ontology from the MeSH graph to develop UDT-RF, aiming to categorize literature into the corresponding cancer hallmarks through text annotation by estimating the information of interest contained. Using the Time Event Ontology (TEO), Chen et al. [64] semantically modeled the time component of Common Data Elements (CDEs) that, in capturing clinical research data, highly benefit from a temporal dimension. For HCC, in addition to developing OntHCC, Messaoudi et al. [44] used it to help in the classification of the staging of tumors that are detected in medical imaging.

#### 5.1.2. Data Integration

A vital part of having large amounts of data in differing repositories and/or originating from various sources is integrating them into a single cohesive semantic representation.

Salvi et al. [11] used a focused ontology to annotate their data from various sources that they have compiled in their relational database concerning Oral Squamous Cell Carcinoma (OSCC). The web-based application LncRNA Ontology was developed by Li et al. [65] from the results of their approach to predict probable functions of most human long non-coding RNAs (lncRNAs). Focusing on reusability and comparison of different sources, Milian et al. [66] developed a method that automatically structures clinical trial eligibility criteria from text. Kim et al. [67] used a graph-based framework that integrates multi-omics data with genomic knowledge in order to improve predictions of clinical outcomes. Wu et al. [68] developed a focused view of the DO from a variety of cancer datasets of various sources in order to enable pan-cancer analysis across datasets. Bona et al. [69] focused on accessibility of non-image data from the Cancer Imaging Archive (TCIA) by using ontologies to integrate it into semantic representations. In their two papers, refs. [53,70] also created a focused ontology, OCRV, but then used it with a data integration pipeline for data in relational databases with the aim of making the semantic relationships explicit and clear across different sources. Hasan et al. [71] developed a prototype of a KG that semantically encodes cancer registry data with the expressed aim of enabling the connection to third-party data to further enable new research. Li et al. [72], on the other hand, constructed a KG by first extracting knowledge triples from available data and then using these to construct a network for healthcare professionals that allows them to traverse this contextualized knowledge. Tao et al. [12] developed a web-based system called Interactive Mapping Interface (IMI) to first map the data dictionary in use by the North American Association of Central Cancer Registries (NAACCR) to the NCIt with the final goal of facilitating the dissemination and reuse of North American cancer registries data. Chen et al. [73] established a consensus knowledge for cancer hallmarks using functional annotations and gene set overlap, again aiming towards enabling the ability to compare data from different sources.

#### 5.1.3. Database Interfaces

One application reported in the articles lies on ontology-based annotations to create user interfaces for databases, where labels of ontology classes and relations allow text annotation. These interfaces are notably useful in dealing with medical data, for integration and querying of different knowledge resources.

Works within this category that have already been mentioned before are Myneni et al. [34] and Esteban-Gil et al. [39] from data annotation, and Milian et al. [66], Hasan et al. [71], and Tao et al. [12] from data integration. Sesen et al. [51] used a lung ontology with the clinical decision support application Lung Cancer Assistant to categorize patients and produce treatment recommendations. González-Beltrán et al. [74] aimed to ease queries over cancer research data, by extending an existing tool, caGrid [75], with additional services, its domain metadata consisting of ontology-based annotations associated with the structural information of each incorporated data source. In lung cancer, circ2GO is a database developed by Lyu et al. [76] that holds information about the functional annotation of circular RNAs by integrating GO information for all genes in their dataset.

#### 5.1.4. Natural Language Processing

Natural Language Processing (NLP) is also a field that can benefit from the use of a standardized organization of knowledge and terms. The works by Milian et al. [66] and Yan et al. [13] have been mentioned in previous sub-categories. In the case of Tapi Nzali et al. [42], the goal was to use their own french CHV of non-experts’ expressions for breast cancer and compare them to biomedical terms used by health care professionals. Directed toward a social scope, Lee et al. [43] created an ontology from a social media crawler and NLP, to evaluate social media data and understand information needs and emotions related to cancer.

**Table 2:** Terminology-focused applications.

Ref	Summary	Ontologies	Data	Tag	Cancer Type
[51]	Ontology for a clinical decision support system to produce treatment recommendations	SNOMED-CT, New ontology	N/A	Database Interface	Lung
[74]	Ontology-based querying for cancer research data	NCIt	N/A	Database Interface	Various
[77]	Mining of genetic marker data in a journal	SNOMED-CT, HUGO	NEJM	NLP	Various
[11]	Automatic translation of NeoMark relational database	BFO, RO, OBI, OGMS, HDO	NeoMark database	Data Integration	OSCC
[15]	Manual identification and inference of associations between breast cancer drugs	New ontology	PharmGKB, NCI	Data Annotation	Breast
[65]	Genome-wide functional predictions of lncRNAs	GO	Gencode, Ensembl, ENCODE project LncRNA Ontology	Data Integration	Various
[66]	Extraction of semantic entities in eligibility criteria and annotation	UMLS	CTG	Data Integration, Database Interface, NLP	Breast
[34]	Development of an ontology-driven survivor engagement framework for mobile apps	FOAF	N/A	Database Interface, Data Annotation	POCS
[67]	Prediction of clinical outcomes from a graph-based approach with multi-omics and genetic data	GO	TCGA	Data Integration	Ovarian
[68]	Development of a focused view within the DO from cancer datasets	DO	COSMIC, TCGA, ICGC, TARGET, IO, EDRN	Data Integration	Various
[39]	Development of a platform for analysis and visualization of data	ICD10, ICD-O-3, TNM staging, SIO, OBI, OQuaRE	NCRI	Data Annotation, Database Interface	Various
[13]	Automatic annotation of cancer hallmarks on biomedical literature	MeSH	N/A	Data Annotation, NLP	Various
[70]	Connection of predictors with cancer survival with a use-case ontology	OCRV	FCDS 2000 U.S. census, BRFSS	Data Integration	Various
[69]	Data integration of several databases with ontologies to enable querying of patient data	DO, UBERON	TCIA, TCGA, LIDC-IDRI, Head-Neck-PET-CT	Data Integration	Various
[78]	Construcion of OCRV based on data analysis needs	NCIt, TEO, ICD-O-3, ICD-9-CM	UF Health CCCA, FCDS, ATSDR, USCB, BRFSS, County Health Ranking & Roadmaps	Data Integration	Various
[64]	Manual representation of semantic temporal components of CDEs	TEO	NCI, caDSR	Data Annotation	Various
[44]	Ontology built following the MethOntology methodology [79]	DICOM	University Hospital of Clermont-Ferrand	Data Annotation	HCC
[42]	Semi-automatic development of CHV for breast cancer	INDC dictionary	N/A	NLP	Various
[71]	KG of cancer registry data, with data analysis and visualization	New ontology	LTR	Data Integration, Database Interface	Various
[43]	Development of an ontology to understand information needs and emotions	LCO, BCO, GCO, SOSW	N/A	NLP	Various
[72]	KGHC is a KG constructed from clinical data available publicly	UMLS	PubMed, UpToDate, CTG, SemMedDB	Data Integration	HCC
[76]	Functional annotation of circRNAs obtained from sequencing lung cell lines	GO	Lung cell lines sequencing data	Database Interface	Lung
[12]	IMI is a web-based system that creates mappings from the NAACCR data dictionary to NCIt	NAACCR data dictionary, NCIt	KCR	Data Integration, Database Interface	Various
[73]	Comparative analysis of cancer hallmark mapping strategies	GO	MSigDB, KEGG, cancer hallmark mapping schemes, TCGA	Data Integration	Various

### 5.2. Semantic-Focused Applications

#### 5.2.1. Formalized Definitions and Axioms: Reasoning with Ontologies

In the works collected, reasoning is applied to the inference of new knowledge from ontologies or error detection is also reported, as summarized in Table 3. The most common way to access and use reasoners in the reviewed papers consisted of using Protégé, an ontology editor, while creating or editing ontologies, due to ease of access [80].

There are works that use reasoners to infer new knowledge from semantically annotated data and/or established rules. Alfonse et al. [81] used FaCT++ to determine the type and stage of a patient’s cancer in order to recommend treatments. Zhu et al. [15] used a rule-based Description Logic (DL) unnamed OWL reasoner to infer additional associations in pathways, drugs, genes and diseases for 18 breast cancer drugs from the ontological representation of the PharmGKB pathway data file. Moreover, using the same ontological representation of PharmGKB, Tao et al. [82] used Pellet to predict new targets for therapy development. Mahmoodi et al. [49] derived association rules from the GCO and patient data using a modified version of an Apriori algorithm, to establish system-wide associations between events in text through large-scale text mining. Barki et al. [52] predicted side effects of treatments for bladder cancer with Pellet. Nicholson et al. [83] used reasoners to signal rule violations in the validation of international rules for multiple primary tumors.

Reasoners can also be used to detect errors in the ontologies or models that have been built. Works by Barki et al. [52], and Nicholson et al. [30,83] were described above. Herrmann et al. [38] aimed at providing a generalizing pattern to classify tumors. Boeker et al. [46] used HermIT DL in their TNM Ontology to evaluate its soundness. Oyelade et al. [14] focused on addressing the issue of vagueness in breast cancer ontology (BCO).

**Table 3:** Semantic-focused applications: reasoning with ontologies.

Ref	Objective	Input Ontologies	Reasoner	Tag	Cancer Type
[81]	Determine cancer type and stage of the patient to recommend treatments	LuCO, BCO, LCO	FaCT++	New Knowledge Inference	Various
[15]	Identification of new indications for existing drugs	New ontology	Automated semantic inference (Protégé)	New knowledge Inference	Breast
[82]	Prediction of new drug targets	New ontology	Pellet (Protégé)	New knowledge Inference	Colorectal
[49]	Extraction of association rules from large datasets on gastric cancer patients	GCO	Apriori algorithm	New Knowledge Inference	Gastric
[38]	Provide a generalizing pattern of more concise definitions to correctly classify all tumor configurations	New ontology	HermiT DL (Protégé)	Error Detection	Various
[46]	Creation of TNM-O	FMA, BioTopLite 2	HermIT DL	Error Detection	Various
[52]	Predict side effects of bladder cancer treatments	New ontology	Pellet (Protégé)	New knowledge Inference + Error Detection	Bladder
[83]	Signal rule violations in a validation process of multiple primary tumors international rules	ICD-O-3	FaCT++, HermiT	New knowledge Inference + Error Detection	Multiple primary tumors
[30]	Facilitate the integrity and maintenance of ENCR core data set	New ontology	FaCT++ (Protégé)	Error Detection	Various
[14]	Minimizing vagueness in the formalization of medical knowledge	DO	Fuzzy DL, HermiT/Pellet (Protégé)	Error Detection	Breast

#### 5.2.2. Mining and Analyzing Multimodal Data with Ontologies

By far the majority of the works reviewed, fall into the category of mining and analyzing, as can be partially observed by Table 4 and the additional 72 gene set enrichment articles not present in it that belong to this category. The use of ontologies in cancer research has undoubtedly opened a new avenue in data analysis, where different methodologies (or combinations of) are used to achieve the most varied goals to derive meaning from large quantities of data.

One of the applications reported in data analysis and mining is semantic filtering [84]. The annotation of data with its semantic concepts enables the use of those same concepts to filter data. Chen et al. [85] used biomedical ontologies to guide a set of sequential filtering steps with the objective of predicting microRNAs related to the regulation of glucocorticoid resistance in the specific case of pediatric acute lymphoblastic leukemia (ALL). In another case, users can use the Semantic Web platform developed by Esteban-Gil et al. [39] to run semantic queries over the annotated data and visualize the results in different ways.

An additional use is similarity measuring [86], where the distance between items is measured by the overlap in meaning, to discern what concepts (and therefore their data) are closer or further apart. For example, Modules and Gene Ontology-based Gene Prioritization, developed by Su et al. [17], uses fuzzy similarity for cancer-related gene prioritization.

One of the main approaches used to analyze large amounts of biomedical data is the employment of ML techniques on data that has been semantically annotated. With the evolution of AI algorithms, researchers have been increasingly able to pose more complex questions and use various methodologies to obtain their answers, which is easily observed from the variety of methods and objectives in the articles reviewed. UMVMO-select is a Unsupervised Multi-View Multi-Objective clustering-based gene selection approach developed by Acharya et al. [87] that uses functional annotation to identify gene markers. Su et al. [88] used an ML method over functionally annotated genetic information to look into the immunofunctionomes of ovarian clear cell carcinoma (OCCC). Chen et al. [64] predicted drug synergy using a deep belief network over genetic expression and an ontological profile of genes built from literature (Ontology Fingerprints). For clinical decision support, Shen et al. [19] outlined an architecture that combines Case-Based Reasoning (CBR) with a Multi-Agent System (MAS) to provide treatment suggestions. [77] used the Multi-threaded Clinical Vocabulary Server (MCVS) NLP engine to mine data related to genetic markers from the New England Journal of Medicine (NEJM), with the aim of further supporting the role of inflammation in cancer. To predict drug targets, Tao et al. [82] used a combination of ontology reasoning with network-assisted gene ranking over an ontology that represents PharmGKB data. Althubaiti et al. [18] used neuro-symbolic feature learning over several ontologies to predict cancer driver genes. Deep GONet, developed by Bourgeais et al. [89], is a self-explainable deep learning model where each biological function is represented by a neuron, that can be used to predict phenotypes. Gao et al. [50] obtained drug inference results from a treatment-based cancer ontology obtained by Bayesian derivation. Comparing the same method with and without ontologies, Min et al. [90] used a rule learning system to predict patients’ ability to perform activities of daily living. Furthermore, to predict cervical cancer cells from cytological tissue images, Divakar et al. [58] used deep neural networks (DNN) on their developed ontology. Salvi et al. [11] used a variety of classifiers—Bayesian networks, artificial neural networks (ANN), support vector machines (SVMs), decision trees and random forests—in a data analysis model of their NeoMark system that holds its own semantic model. By comparing several different models, Yan et al. [13] reached an approach that outperforms the others that uses ontological features with a combined use of United Decision Trees and Random Forest algorithms. González-Beltránet al. [74] developed a system for ontology-based queries over the caGrid infrastructure than can be reused with other service-oriented and model-driven infrastructures. Xi et al. [91] leverages KG embeddings for tolerating missing data from breast cancer clinical ultrasound reports. Using graph attention networks (GAT), Zhang et al. [92] developed a method for real-time inference on a lung KG, using a new ontology.

However, in the end, the most common approach to the use of ontologies in the analysis of biomedical data was the application of GO in Gene Set Enrichment Analysis (GSEA) [16,53,73,93,94,95,96,97,98,99,100,101,102,103,104,105,106,107,108,109,110,111,112,113,114,115,116,117,118,119,120,121,122,123,124,125,126,127,128,129,130,131,132,133,134,135,136,137,138,139,140,141,142,143,144,145,146,147,148,149,150,151,152,153,154,155,156,157,158,159]. GSEA statistically compares set of genes that share biological characteristics and interprets their expression data in light of on whether they differ across defined phenotypes [160] and as such is commonly used in biomedical research to, for example, establish candidate genes for further studies.

**Table 4:** Semantic-focused applications: mining and analyzing multimodal data with ontologies.

Ref	Objective	Method	Input Ontologies	Input Data	Tag	Cancer Type
[77]	Mining of genetic marker data in a journal	MCVS NLP engine	SNOMED CT, HUGO	NEJM	ML	Various
[74]	Ontology-based querying for cancer research data	Construction of a OWL Generation facility	NCIt	caGrid	ML	Various
[11]	Represent the project domain and link the NeoMark data to other domains	Bayesian Networks, ANN, SVMs, Decision Trees, Random Forests	BFO, RO, OBI, OGMS, HDO	N/A	ML	OSCC
[50]	Cancer reclassification and drug inference	Vazquez Bayesian clustering algorithm	N/A	HemOnc.org	ML	Various
[19]	Ontological application in Clinical Decision Support	CBR and MAS	UML	Patient Health Records	ML	Gastric
[82]	Prediction of new drug targets	KEGG functional PharmGKB drug annotation. Network neighborhood modeling ranking	New ontology, ATC	PharmGKB, GAD, CGC, OMIM, NCI, DrugBank, TTD	ML	Colorectal
[39]	Design of a semantic model for local cancer registries	Ontology-driven search filters and aggregates properties of interest	ICD10, ICD-O-3, TNM staging, SIO, OBI, OQuaRE	NCRI	Filtering	Various
[90]	Discover patterns related to the patients’ ability to perform daily living activities	AQ21—multi-task ML and data mining system	UMLS	Surveillance, Epidemiology, and End Results—Medicare HOS	ML	Various
[13]	Automatic annotation of cancer hallmarks on biomedical literature	United Decision Tree and Random Forest	MeSH	Pubmed abstracts	ML	Various
[85]	Prediction of microRNA related to glucocorticoid resistance	Manual background literature search. Semantic searches in resulting subset	OMIT, NCRO, MeSH	PubMed	Filtering	Pediatric ALL
[17]	Cancer-related gene prioritization	Fuzzy similarity	GO	GSEA website, TCGA, SNP4Disease	Similarity	PAC, Breast
[161]	Predict drug synergy in cancer treatment	Stacked Restricted Boltzmann machine	GO, Ontology Fingerprints	AstraZeneca-Sanger Drug Combination Prediction Challenge, GDSC, KEGG	ML	Various
[18]	Identification of cancer driver genes with role distinction	Neuro-symbolic deep learning on semantic knowledge representation on genetic information	CMPO, GO, MP	Uniprot, MGI database, Mutational Cancer Drivers Database, CPD	ML	Naso-pharyngeal, Colorectal
[87]	Identification of relevant, expression data non-redundant cancer gene markers	Unsupervised Multi-View Multi-Objective clustering	GO	Gene expression datasets from own lab	ML	Prostate, DLBCL, FL
[58]	Predict cervical cancer cells from cytological tissue images	DNN	New ontology	hospital cervical cancer data, kaggle data repository	ML	Cervical
[88]	Complement system role inference from immunofunctionome analysis	SVMs	GO	GEO database	ML	OCCC
[89]	Cancer detection based on gene expression data	Multilayer Perceptrons	GO	Affymetrix HG-U133Plus2 chip arrays, TCGA	ML	Various
[91]	Tolerating data missing in breast cancer diagnosis from clinical ultrasound reports	KG embeddings	BI-RADS	Ultrasound reports	ML	Breast
[92]	Real-time inference on a lung KG	GAT	New ontology	KEGG, Uniprot, DrugBank, TCGA	ML	Lung

Of the 141 papers selected in this systematic review, 72 employed gene set enrichment in some manner. Of these, 21 only used GO, and 48 used it in conjunction with other resources, of which Kyoto Encyclopedia of Genes and Genomes (KEGG) pathway database was more common with 45 articles, followed by REACTOME pathway database with 3. Of this application, we have the example of Tian et al. [131] that profiled the transcriptome of gastric cancer patients and used the enrichment to confirm the annotation of genes with digestive system process, secretion and digestion. She et al. [109] used GO and KEGG in an enrichment analysis with the overall objective of finding the importance of C reactive protein and its interactors in HCC. Moreover, developing research in the same cancer, Agioutantis et al. [16] also used enrichment with both GO and REACTOME in their pursuit of deciphering molecular heterogeneity and drug responsiveness by exploring the molecular diversity of tumors and drug sensitivity. No table is provided for this type of use since the methodology is standardized.

## 6. Conclusions

Over the last two decades, ontologies gained traction in biomedical research in general, and cancer research in particular, enabling FAIR data (findability, accessibility, interoperability and reusability) [162], supporting data integration and analysis, and facilitating data interpretation and data mining. Presently, we are witnessing the emergence of the knowledge graph paradigm, whereby large volumes of heterogeneous data are brought together under a single holistic ontological knowledge model. Yet, there are still a number of open challenges to the development and application of ontologies and knowledge graphs for cancer research.

One major challenge lies in reusing existing ontologies. With over 800 biomedical ontologies publicly available in BioPortal [3], most biomedical subjects are covered by one or more ontologies, and it might seem foolish not to reuse them. However, the fact that there are so many ontologies and many overlap in domain makes it difficult to navigate the ontology landscape and select which ones to reuse. Moreover, many ontologies were typically developed with a singular purpose in mind, and have a particular perspective on the domain they model which may be unsuited for other purposes. This means that additional care is needed when selecting ontologies to reuse, to make sure that their perspective on the domain is compatible with the new use case. Last but not least, it may be the case that existing ontologies are no longer actively maintained and kept up to date, which in a dynamic domain like biomedicine, will render them useless in a short time span. Ultimately, it may very well be that no existing ontology is compatible with or usable in the new use case, and that a new ontology must be developed, which indeed is the main reason why there are presently so many ontologies. Thus, to avoid perpetuating the problem, new ontologies should be designed circumspectly, taking into account possible other applications within their specific domain [30].

Another challenge lies in the disconnection between data and ontologies, due to the fact that, in the large majority of cases, biomedical ontologies do not include data. In fact, few biomedical ontologies were designed with the prospect of directly encoding data, as the biomedical research community has, for the most part, viewed ontologies merely as abstract knowledge models used for classification or at best annotation of data, with the data kept in relational databases or even data files. This is tied to the reusability challenge, as existing ontologies may not be reusable for use cases such as constructing knowledge graphs if they are unsuited to being instantiated. Furthermore, it means that constructing biomedical knowledge graphs to support cancer research requires (semi-)automated approaches to integrating the data with the knowledge model, which, considering the variety and heterogeneity of relevant biomedical data sources, can be burdensome [163]. However, as the knowledge graph paradigm becomes more popular, we may witness a shift in the biomedical community towards storing data in graph databases rather than relational databases.

Tied to the two previous challenges is the challenge of integrating multiple ontologies, a necessity for constructing holistic knowledge graphs for cancer research, due to the multidisciplinarity of the domain. Although there are comprehensive ontologies on cancer (e.g., NCIt), available data is often connected to more specialized ontologies (e.g., GO, MeSH), eliciting the need to integrate them. The problem is that, due to their different perspectives, overlapping ontologies may be semantically irreconcilable [164], which may impede their joint use. Thus, the costs of reusing existing ontologies may outweigh their benefits, prompting the development of an independent ontological knowledge model for a knowledge graph, ideally with mappings to existing ontologies to ensure interoperability and facilitate data integration.

The benefits of developing holistic knowledge graphs that integrate all the data relevant for cancer research are deeply tied to the potential of AI approaches to unlock knowledge conducive to better diagnostics or treatments. Knowledge graphs can serve as sources of background knowledge to AI approaches, compensating for missing values in the data, they can support image classification and NLP approaches to enrich image or textual data, which in turn can improve the performance of AI approaches relying on that data, and they provide a means to afford explainability to AI approaches [165], tackling the black-box problem of state-of-the-art AI methods.

The immense potential of ontologies and the knowledge graph paradigm to support cancer research data management and analysis is increasingly recognized by the oncology research community as an essential building block of the P4 medicine vision (preventative, predictive, personalized and participatory).

## Data Availability

Not applicable.

## Abbreviations

The following abbreviations are used in this manuscript:

AI	Artificial Intelligence
ATC	Anatomical Therapeutic Chemical
ATSDR	Agency for Toxic Substances and Disease Registry
ALL	Acute Lymphoblastic Leukemia
ANN	Artificial Neural Network
BCFO	Breast Cancer Fuzzy Ontology
BCO	Breast Cancer Ontology
BFO	Basic Formal Ontology
BRFSS	Behavioral Risk Factor Surveillance System
BTL2	BioTopLite 2
caDSR	Cancer Data Standards Repository
CBR	Case-Based Reasoning
CCL	Cancer Cell Ontology
CCTOO	Cancer Care Treatment Outcome Ontology
CDEs	Common Data Elements
CGC	Cancer Gene Census
CHV	Consumer Health Vocabulary
CL	Cell Ontology
CLO	Cell Line Ontology
CMPO	Cellular Microscopy Phenotype Ontology
COBRA	COnsortium for BRachytherapy data Analysis
COnQueSt	Cancer Ontology Querying System
CPD	Cellular Phenotype Database
CTCAE	Common Terminology Criteria for Adverse Events
CTG	ClinicalTrials.gov
DICOM	Digital Imaging and Communications in Medicine
DL	Description Logic
DLBCL	Diffuse Large B Cell Lymphoma
DO	Disease Ontology
EFO	Experimental Factor Ontology
ENCR	European Network of Cancer Registries
ENCR core-data	European Cancer-Registry core-data ontology
FCDS	Florida Cancer Data System
FL	Follicular Lymphoma
FMA	Foundational Model of Anatomy
FOAF	Friend of a Friend ontology
FOORC	Fuzzy Ontology for Obesity-Related Cancer
GAD	Genetic Association Database
GCO	Gastric Cancer Ontology
GDSC	Genomics of Drug Sensitivity in Cancer
GO	Gene Ontology
HDO	Human Disease Ontology
HCC	Hepatocellular Carcinoma
HOS	Health Outcomes Survey
HUGO Gene Nomenclature	Human Genome Organization Gene Nomenclature
ICD-9-CM	International Classification of Diseases Ninth Revision Clinical
	Modification
ICD-O-3	International Classification of Disease for Oncology 3rd edition
IMI	Interactive Mapping Interface
IOBC	Interlinking Ontology for Biological Concepts
KCR	Kentucky Cancer Registry
KEGG	Kyoto Encyclopedia of Genes and Genomes
KG	Knowledge Graph
LCO	Liver Cancer Ontology
LCKGO	Lung Cancer Knowledge Graph Ontology
LINCS	Library of Integrated Network-based Cellular Signatures
lncRNAs	long non-coding RNAs
LOINC	Logical Observation Identifier Names and Codes
LTR	Louisiana Tumor Registry
LuCO	Lung Cancer Ontology
MAS	Multi-Agent System
MCVS	Multi-threaded Clinical Vocabulary Server
MedDRA	Medical Dictionary for Regulatory Activities
MeSH	Medical Subject Headings
MGI	Mouse Genome Informatics
ML	Machine Learning
MP	Mammalian Phenotype ontology
MuEVo	Multi-Expertise Vocabulary
NAACCR	North American Association of Central Cancer Registries
NCI	National Cancer Institute
NCIt	National Cancer Institute Thesaurus
NCRI	National Cancer Registry Ireland
NCRO	Non-Coding RNA Ontology
NEJM	New England Journal of Medicine
NLP	Natural Language Processing
OBDA	Ontology-Based Data Access
OBI	Ontology for Biomedical Investigators
OCCC	Ovarian clear cell carcinoma
OCRV	Ontology for Cancer Research Variables
OCRSEV	Ontology of Cancer Related Social-Ecological Variables
OD-ATTEST	Ontology for the Documentation of vAriable selecTion and daTa
	sourcE Selection and inTegration
ODVDS	Ontology for Documentation of Variable and Data Source
OGMS	Ontology of General Medical Science
OIE	Open Information Extraction
OMIM	Online Mendelian Inheritance in Man
OMIT	Ontology for MicroRNA Target
OntHCC	Ontology of Hepatocellular Carcinoma
OQuaRE	Ontology Quality Evaluation Framework
OSCC	Oral Squamous Cell Carcinoma
OWL	Web Ontology Language
PAC	Prostatic Adenocarcinoma
POCS	Profile Ontology for Cancer Survivors
QHIO	Quantitative Histopathological Imaging Ontology
RO	Relation Ontology
ROS	Radiation Oncology Structures
SCRS	Semantic Cancer Registry System
SEER-MHOS	Surveillance, Epidemiology, and End Results—Medicare Health
	Outcomes Survey
SIO	Semanticscience Integrated Ontology
SKOS	Simple Knowledge Organization System
SNOMED CT	Systematized Nomenclature of Medicine Clinical Terms
SNOMEDint	SNOMED International
SOSW	Sentiment Ontology for Social Web
SVMs	Support Vector Machines
SWIT	Semantic Web Integration Tool
TCGA	The Cancer Genome Atlas
TEO	Time Event Ontology
TNM	Tumor–Node–Metastasis
TNM-O	Tumor–Node–Metastasis Ontology
TOCSOC	Temporal Ontology for Comparing the Survival Outcomes
TTD	Therapeutic Target Database
UMLS	Unified Medical Language System
USCB	United States Census Bureau
